# Ultrafast Continuum IR Generation and Its Application in IR Spectroscopy

**DOI:** 10.3390/ijms232113245

**Published:** 2022-10-31

**Authors:** Chaiho Lim, Kwanghee Park, Yeongseok Chae, Kyungwon Kwak, Minhaeng Cho

**Affiliations:** 1 Center for Molecular Spectroscopy and Dynamics, Institute for Basic Science (IBS), Korea University, Seoul 02841, Korea; 2 Department of Chemistry, Korea University, Seoul 02841, Korea

**Keywords:** ultrafast IR spectroscopy, continuum IR, filamentation

## Abstract

The spectral range of femtosecond time-resolved infrared spectroscopy is limited by the bandwidth of mid-IR pulses (100~400 cm^−1^) generated from the combination of Ti:Sapphire amplifier, Optical Parametric Amplifier (OPA), and Difference Frequency Generation (DFG). To overcome this limitation, we implement a compact continuum mid-IR source producing ultrafast pulses that span the frequency range from 1000 to 4200 cm^−1^ (from 10 to 2.4 μm), which utilize the mixing of fundamental, second-harmonic, and third-harmonic of 800 nm pulse in the air. After building an IR spectrometer with continuum IR and a monochromator, we found that the distortion of the measured IR spectrum originated from the contamination of higher-order diffraction. We used bandpass filters to eliminate the higher-order contributions and correct the measured IR spectrum. We further characterized the spectral properties of fundamental, second-harmonic, and third-harmonic fields after the plasmonic filamentation process, which helps to improve the efficiency of the continuum IR generation. Using the generated continuum IR pulses, we measured the IR absorption spectrum of a water–benzonitrile mixture, which was found to be consistent with the spectrum obtained with a commercial FT-IR spectrometer. The present work will be useful for the efficient generation of continuum IR pulses for IR pump-probe and two-dimensional IR spectroscopy experiments in the future.

## 1. Introduction

The time-resolved ultrafast nonlinear infrared (IR) spectroscopy has been used to study chemical reactions and solvent dynamics such as energy transfer, proton hopping, protein denaturation, and chemical exchange processes in condensed phases. A chemical reaction is a process during which substrates are converted into products with the possibility of going through various transition states and intermediates. The reaction’s rate, selectivity, and outcome are determined by the properties of these transition-state species, reaction intermediates, and reaction conditions (e.g., temperature, pH, etc.).

A vast number of reactions of great practical, technological, and biological importance, including the primary events in vision such as the isomerization of the retinal chromophore in rhodopsin and bacteriorhodopsin, oxygen dissociation and association in hemeproteins, transmembrane electron transport in photosynthetic bacterial reaction centers, and solvent and ligand dynamics in the electrolyte solvation sheath of a lithium-ion battery, involve ultrafast molecular dynamics of solvent molecules or protein residues in the vicinity of reactive species. Owing to the advancements in ultrafast laser technology, it becomes possible to observe short-lived intermediates and transition-state species by monitoring the characteristic IR-active modes or site-specifically incorporated IR probes in real time. The technological improvements of time-resolved IR spectroscopies in detection sensitivity, experimental stability and reliability, temporal resolution, and wavelength tunability enabled the measurement of reaction dynamics on the picosecond and even femtosecond time scales.

The ultrafast IR spectroscopy uses a mid-IR laser pulse generated from Ti:Sapphire regenerative amplifier laser combined with OPA and DFG. This approach is, however, of limited use because of the narrow phase-matching condition of nonlinear crystals involved in each step. The mid-IR laser pulse has a relatively narrow bandwidth (<400 cm^−1^)—note that the fingerprint molecular vibrational spectrum spans from 400 to 4000 cm^−1^. A few research groups have attempted to overcome this problem of narrow bandwidth by developing continuum IR sources [1,2,3,4,5,6,7]. Fuji et al. generated a femtosecond continuum IR pulse the spectrum of which covers the wavenumber range from 200 to 5500 cm^−1^ using a four-wave difference-frequency mixing process in air plasma, where they used fundamental (800 nm) and second harmonics (400 nm) of Ti:sapphire-based ultrafast laser output [1,4,5,6]. Petersen and Tokmakoff used fundamental (800 nm), second harmonics (400 nm), and third harmonics (266 nm) of 800 nm laser pulses to generate continuum IR pulses covering 400 to 3300 cm^−1^ [2]. They could further implement the continuum IR generation scheme to carry out time-resolved nonlinear IR spectroscopy experiments [8,9,10,11,12]. However, due to the broad spectral bandwidth of continuum IR pulses, the detection of nonlinear IR signals using a monochromator is inevitably contaminated by higher-order diffraction stray light in the short wavelength (high frequency) region when the long wavelength (low frequency) signal is detected. Consequently, it is difficult to measure the frequency-resolved pump-probe or 2D-IR spectra accurately.

Here we report the implementation of the four-wave mixing difference frequency generation by ambient air. To improve the stability and efficiency of continuum IR generation, we examined the spectral feature of each pulse involved in the four-wave mixing process before and after plasma generation. Using the generated continuum IR pulse, we measured the IR spectrum of water-benzonitrile mixed solutions from 1000 to 4000 cm^−1^ and compared it with the spectrum from the conventional FT-IR spectrometer. The lower frequency part of the IR spectrum was contaminated by the higher-order diffraction, but this problem was overcome by measuring the low-frequency and high-frequency regions separately using two long-pass filters. Thus, we believe that the continuum IR pulses are ready to be used as probe pulses for time-resolved IR spectroscopy.

## 2. Results and Discussion

**Figure 1:**
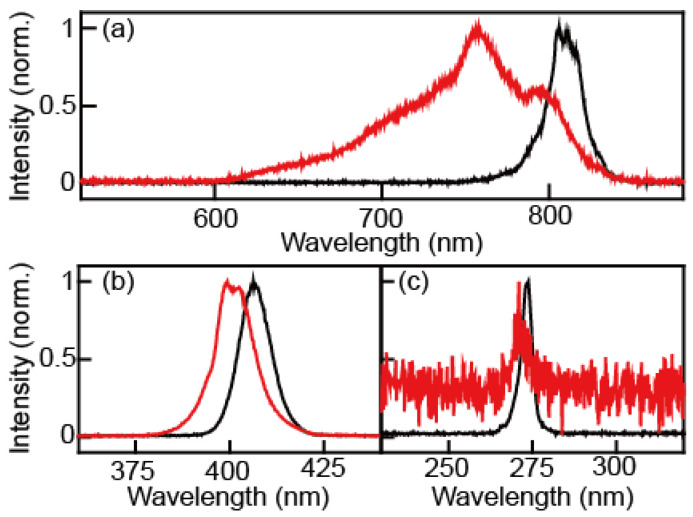
Spectral changes after plasma generation. (**a**) Fundamental source before (black) and after (red) the air plasma. (**b**) Second harmonic source before (black) and after (red) the air plasma. (**c**) Third harmonic source before (black) and after (red) the air plasma.

In the process of continuum IR generation, we observed the spectral changes of the fundamental, second-harmonic, and third-harmonics pulses, as shown in Figure 1. The spectra and pulse energies of the fundamental, second-harmonic, and third-harmonic beams were measured before and after the air plasma generation using appropriate harmonic separators and filters, respectively. A UV/Vis spectrometer (Flame, Ocean Optics) was used to measure the spectrum. The fundamental beam was separated by the harmonic separator (042-4805, Eksma, Vilnius, Lithuania) and the 610 nm long-pass filter (FSQ-RG610, Newport). In the case of the second-harmonic field, the harmonic separator (042-4805, Eksma) and 468 nm short-pass filter (FF01-468/SP-25, Semrock, West Henrietta, NY, USA) were used to separate it from other lights. The 468 nm short-pass filter blocks the third-harmonic and the fundamental because only light with wavelengths shorter than 330 nm was allowed to pass through the filter. For the third harmonic, the harmonic separator (042-2485, Eksma) and 311 nm short pass filter (FF01-311/SP-25, Semrock) were used to separate it from the other radiations. The center peak and full width at half-maximum (FWHM) of the fundamental beam were 805.2 nm and 25.6 nm before the generation of the air plasma and 750.4 nm and 98.3 nm after passing the air plasma, respectively. The peak wavelength and FWHM of the second-harmonic were 402.6 nm and 10.3 nm before the air plasma and 397.1 nm and 10.9 nm after the air plasma, respectively. Those of the third-harmonic were 269.1 nm and 4.3 nm before the air plasma and 267.3 nm and 5.7 nm after the air plasma, respectively (Table 1). The measured spectra of pulses before and after the air plasma filamentation indicate that the plasma makes the beams blue-shifted and spectrally broadened. These experimental observations were explained in terms of self- and cross-phase modulations of ultrafast laser pulse [1,13,14].

The fundamental, second-harmonic, and third-harmonic pulses show quite different blue shifting and broadening behaviors, as shown in Table 1. Only the fundamental beam is significantly blue-shifted (~55 nm) and broadened (~4 times) after its interaction with air plasma. Such plasma-induced spectral blueshift is proportional to the extent of optical field-induced ionization and interaction length. In the present experiment, three beams overlap spatially and temporarily, so we could assume that all three beams pass through the same plasma medium. However, the differences in the intensity of the three beams cause variations in the extent of filamentation, which is closely related to the interaction length. The observed decrease in blueshift (i.e., 55 nm, 5.5 nm, and 1.8 nm for the fundamental, second-harmonic, and third-harmonic, respectively) can be understood by noting that the filamentation is a nonlinear process and the three beam intensities are quite different from one another. From the measured center wavelengths of the three beams, it is clear that the continuum IR beam is generated by four-wave mixing processes involving either $\omega_{\mathrm{fund}}+\omega_{2\mathrm{snd}}-\omega_{3\mathrm{trd}}\to \omega_{\mathrm{IR}}$ transition or $2\omega_{\mathrm{fund}}-\omega_{2\mathrm{snd}}\to \omega_{\mathrm{IR}}$ transition. Unfortunately, it is not possible to determine which transition pathway is dominant.

The pulse energy of the fundamental beam was measured with a pyroelectric joulemeter (J-25MT-10kHz, Coherent, Santa Clara, CA, USA), and they were 0.729 mJ and 0.462 mJ before and after the air plasma. The pulse energy of the second harmonic beam was measured by a pyroelectric joulemeter (J-10MB-LE, Coherent), and it was 0.287 mJ and 4.6 μJ before and after the air plasma. The pulse energy of the third-harmonic field before the air plasma was measured to be 2.8 μJ, and that after the air plasma was lower than the detection limit (300 nJ) of the pyroelectric joulemeter (J-10MB-LE, Coherent). Although we used a short-pass filter to selectively measure the third-harmonic pulse energy, still a considerable amount of fundamental beam passed through the spectrometer. Thus, we corrected the contribution of the fundamental beam by measuring its energy using an additional long-pass filter. The conversion efficiency of the third harmonic generation was found to be about 0.2% when the position and angles of crystals were optimized to maximize the intensity of the continuum IR, where the maximum conversion efficiency of the third harmonic generation is ~8%. It implies that the intensity of the continuum IR does not only depends on the intensity of the third-harmonic pulse. In fact, it should be noted that the efficiency of continuum IR generation is strongly affected by the correlation between the polarization states of involved beams instead of the pulse energy of the third harmonic. Indeed, the intensity of continuum IR generation could be maximized when the difference between the polarization states of the beams was minimized [2]. The pulse energy of the continuum IR could not be measured using the pyroelectric joulemeter (J-10MB-LE, Coherent) because it is smaller than the detection limit of 500 nJ. The low efficiency of continuum IR compared to that estimated from the energy loss of fundamental, second-harmonic, and third-harmonic beams implies that most of the energy loss is due to the ionization of the air, not to the continuum IR generation. The intensity stability of the continuum IR pulse was 24% (see details in Supplementary Note S1 and Figure S1).

**Figure 2:**
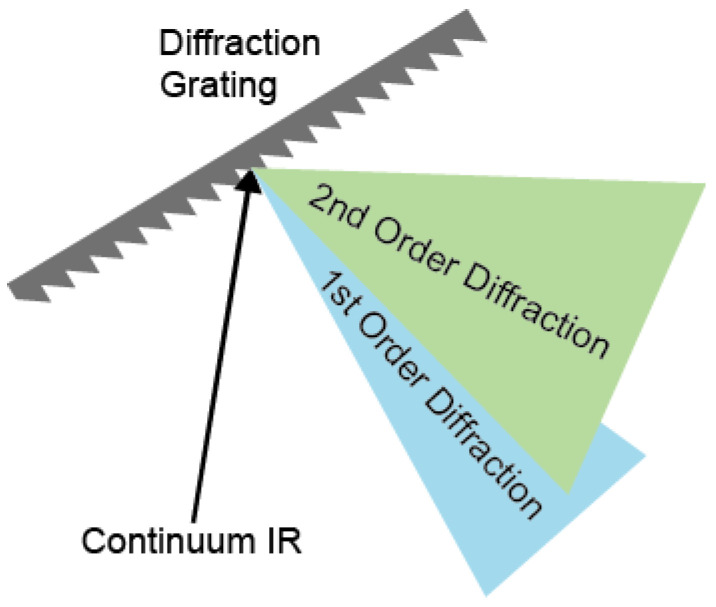
Schematic representation of the diffraction grating for spectral analysis of continuum IR beam.

**Figure 3:**
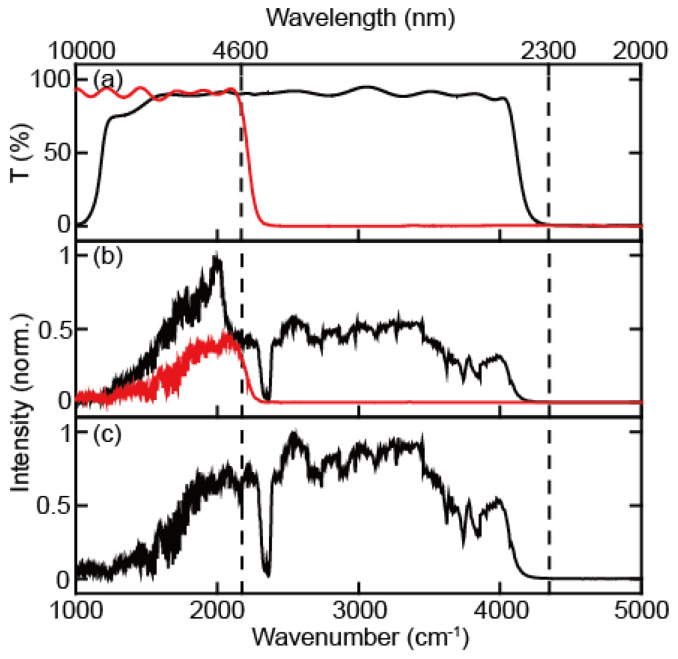
(**a**) Transmittance spectrum of 2.4 μm IR long-pass filter (black) and 4.5 μm IR long-pass filter (red). (**b**) Continuum IR spectra obtained with 2.4 μm IR long-pass filter (black) and 4.5 μm IR long-pass filter (red). (**c**) Corrected continuum IR pulse spectrum, where the spectrum under and over 4.5 μm are obtained with 4.5 μm and 2.4 IR long-pass filter, respectively.

The spectrum of continuum IR pulse was characterized using a monochromator with 100 grooves/mm density diffraction grating (IHR320, Horiba, Kyoto, Kyoto) and liquid nitrogen-cooled MCT array detector (MCT-10-128, InfraRed associates, Stuart, FL, USA). The first-order diffracted beam was typically used for frequency-resolving the spectrum. However, in the case of broadband pulses, higher-order diffracted stray light is not negligible, resulting in a spectral distortion when detecting the long wavelength (low frequency) part of the spectrum (Figure 2). To avoid contamination by the stray light originating from the higher-order diffraction beam, we measured the full spectrum using two filters in two different frequency regions (Figure 3a,b). The long-pass filters of 2.4 μm and 4.5 μm (68–653, 68–655, Edmund) were used for the spectral ranges from 2.3 μm to 4.6 μm and from 4.6 μm to 10 μm, respectively. The continuum IR spectrum with the long-pass filter covers the region between 1000 cm^−1^ and 4000 cm^−1^, and the peak wavenumbers and FWHM are 2838 cm^−1^ and 2124 cm^−1^. This continuum IR pulse is suitable for studying almost the whole molecular fingerprint region (Figure 3c). Since the spectrum measurements were held without purging dry air or N_2_ gas, the peaks in the spectrum of the continuum IR pulse are due to the water vapor (1300~2000 cm^−1^ and 3500~4000 cm^−1^) and the carbon dioxide (~2350 cm^−1^) absorption. However, the intensity of our continuum IR pulse in the low-frequency region (<1500 cm^−1^) decreases rapidly with decreasing wavenumbers not only because the efficiency of diffraction grating used (100 groove density optimized at 6 μm) is low but also because the cut-off frequency of our MCT detector is 8 μm with low detectivity under 1250 cm^−1^.

**Figure 4:**
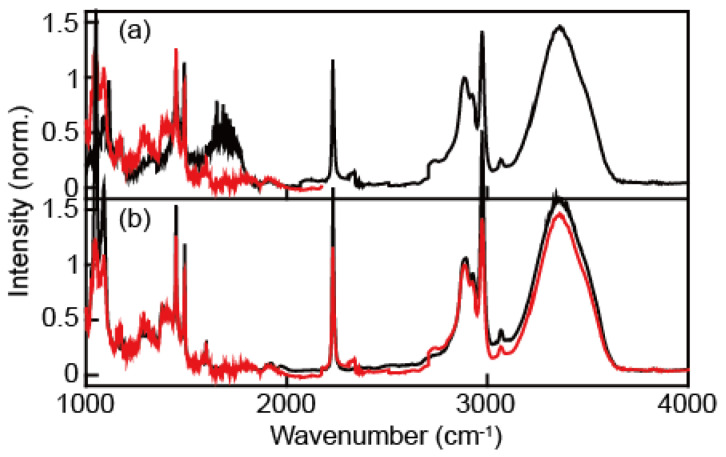
(**a**) The IR absorption spectra of water:benzonitrile (=1:1, volume ratio) solution measured using our continuum IR pulse obtained with a monochromator and a 2.4 μm IR long-pass filter (black) or 4.5 μm IR long-pass filter (red). (**b**) Comparison between the FTIR absorption spectrum (black) and that obtained with a continuum IR pulse (red).

To confirm whether our correction procedure to obtain the full continuum IR pulse (Figure 3c) works, we compared the absorption spectrum of a water and benzonitrile mixture obtained with our continuum IR pulses and a commercial FT-IR spectrometer. In the absorption spectrum of the water and benzonitrile mixture (Figure 4), a broad peak at around 1800 cm^−1^ (black line, Figure 4a) appears only when a 2.4 μm long-pass filter was used. This peak wavelength is exactly twice as large as that of 3500 cm^−1^, which corresponds to the water OH stretching vibration. Therefore, the peak at 1800 cm^−1^ in the spectrum (black line, Figure 4a) originates from the second-order diffraction signal, which is an undesirable contribution to the absorption spectrum. The IR absorption spectrum (red line, Figure 4b) obtained with our continuum IR pulses and 2.4 μm and 4.5 μm long-pass filters is in excellent agreement with that obtained with a commercial FTIR spectrometer. All the peak positions are consistent with each other. However, the absorbances are slightly different from one another, especially when the absorbance is larger than 1. Nonetheless, even though the intensity of the low-frequency (<1500 cm^−1^) component in our continuum IR pulses is weak, the absorption spectrum of the water and benzonitrile mixture is well-matched with the FTIR spectrum.

## 3. Materials and Methods

**Figure 5:**
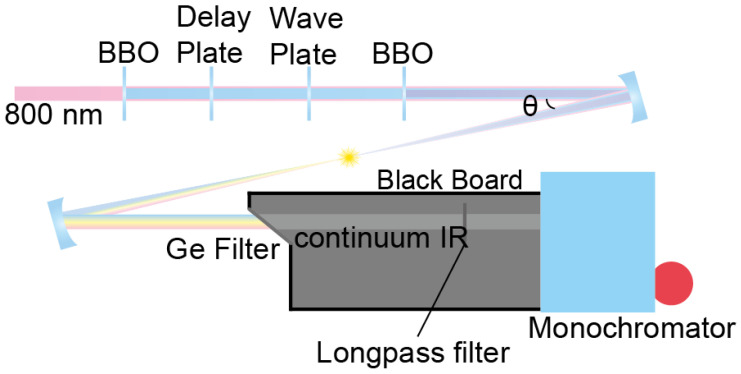
Continuum IR generation setup based on plasma generation. The captured photo of the setup can be seen in Figure S2.

A similar optical setup for the difference frequency generation via four-wave mixing in the air has been reported previously [2]. We use 1.5 mJ, 1 kHz, 25 fs, 800 nm pulse from Ti:Sapphire regenerative laser (Legend Elite Duo, Coherent), which is collimated to maintain its beam size at 5 mm in diameter, which was measured with the knife-edge method, by a pair of concave and convex mirrors. This collimated pulse is sent through four nonlinear crystals in series: (i) a frequency-doubling crystal: type 1 barium borate (BaB_2_O_4_, BBO), 10 mm × 10 mm × 0.2 mm, θ = 29.2°; (ii) a delay plate: calcite window; (iii) zero-order waveplate: single-crystal quartz λ/2 at 800 nm and λ at 400 nm; (iv) a frequency-tripling crystal: Type-1 BBO, 10 mm × 10 mm × 0.05 mm, θ = 44.3° (FK-800050-10 femto kit, Eksma), as shown in Figure 5 schematically. The second- (400 nm) and third-harmonic (266 nm) pulses are generated via frequency-doubling and -tripling of the fundamental (800 nm), respectively. All three beams are temporally and spatially overlapped at the focusing point after the four crystals. The exact position and angle of each crystal are adjusted to optimize the continuum IR intensity and spectrum. The three beams are focused by a 5-cm focal length concave mirror (custom order; reflectance: 98% at 266 nm, 99% at 400 nm, 99% at 800 nm, Eksma), which generates air plasma and continuum IR pulses simultaneously. The intensity of the continuum IR beam depends on the angle of incidence (θ) between the concave mirror and propagating beam (Figure 5), but the spectrum is little affected by the angle. The intensity of the continuum IR beam could be enhanced as the incidence angle was reduced until the optics blocked the laser path. The angle of incidence affects the spatial overlap of focused pulses due to coma aberration. The continuum IR pulse was collimated by a silver concave mirror and separated from other pulses using 3–12 μm AR-coated germanium IR filter (83349, Edmund). Unwanted non-directional stray light is also generated from the air plasma and directed to the detector. Thus, it is important to separate the air plasma generation part from the monochromator and detector with blackboards to block stray light generated from the filamentation. Finally, we were able to obtain a continuum mid-IR spectrum using a monochromator and mercury cadmium telluride (MCT) array detector. While the FTIR spectrometer acquisition speed is limited by scanning time to obtain the interferogram, the IR spectrum using a continuum IR pulse can be obtained with a monochromator and an array detector. In particular, the IR absorption peaks within a frequency range covered by a grating can be obtained at a single shot without moving the monochromator.

## 4. Conclusions

To generate continuum IR pulses, we used collinearly aligned nonlinear crystals to generate the second-harmonic and third-harmonic beams of the fundamental (800 nm) beam from a Ti:Sapphire regenerative amplifier. The pulse energies and spectra of the fundamental, second-harmonic, and third-harmonic beams, which all contribute to the generation of broadband IR pulses, were measured before and after the air plasma, which acts as the nonlinear (four-wave-mixing) optical medium for the continuum IR generation. The blue-shifting and spectral broadening of all three beams were characterized and correlated with the efficiencies of air plasma formation, i.e., filamentation and continuum IR generation. To build an IR spectrometer with continuum IR sources, we showed that the stray light from higher-order diffracted beams from the grating should be eliminated using a long-pass filter. We demonstrated that this correction step was crucial for the development of a single-shot IR spectrometer and future applications of this continuum IR beams in time-resolved nonlinear IR spectroscopy utilizing a monochromator for spectral interferometric detection of nonlinear IR signals. Here, we showed that the IR spectrometer with broadband IR pulses provided the absorption spectrum of water and benzonitrile mixed solutions, which is in good agreement with that obtained with an FTIR spectrometer.

## Figures and Tables

**Table 1:** The peak wavelengths and FWHMs of the fundamental, second-harmonic, and third-harmonic fields measured before and after they interact with filamentation-forming air plasma.

	Center Peak (nm)		FWHM (nm)	
	Before	After	Before	After
Fundamental	805.2	750.4	25.6	98.3
Second Harmonic	402.6	397.1	10.3	10.9
Third Harmonic	269.1	267.3	4.3	5.7

## Supplementary Materials

The following supporting information can be downloaded at: https://www.mdpi.com/article/10.3390/ijms232113245/s1.
