# Supplementary material for: Ultrafast Continuum IR Generation and Its Application in IR Spectroscopy
Source: Int J Mol Sci. 2022 Oct 31;23(21):13245. doi: 10.3390/ijms232113245 (PMC9659240; doi:10.3390/ijms232113245)
Supplement: Supplementary file 1 [file ijms-23-13245-s001.zip › ijms-2006580-supplementary.pdf]

Supporting Information

Ultrafast Continuum IR Generation and Its Application in IR Spectroscopy

Chaiho Lim [1,2], Kwanghee Park [1,2], Yeongseok Chae [1,2], Kyungwon Kwak [1,2,*] and Minhaeng Cho [1,2,*]

[1] Center for Molecular Spectroscopy and Dynamics, Institute for Basic Science (IBS), Seoul 02841, Korea

[2] Department of Chemistry, Korea University, Seoul 02841, Korea

* Correspondence: mcho@korea.ac.kr, kkwak@korea.ac.kr

Supplementary Note S1. The intensity stability of continuum IR pulse

We estimated the intensity stability from the spectrum of continuum IR pulse. The intensity stability was defined as the percent ratio of the noise and the intensity average,

$$\text{Intensity Stability} = \frac{\sigma_N}{\bar{I}} \times 100\ \% \tag{S1}$$

where $\bar{I}$ is the averaged intensity and σ_N is the noise defined as,

$$\sigma_N = \sqrt{N-1}\sqrt{\frac{(I-\bar{I})^2}{n-1}} \tag{S2}$$

where I is the raw intensity, n is the number of intensity data, and N is the number of laser pulse.

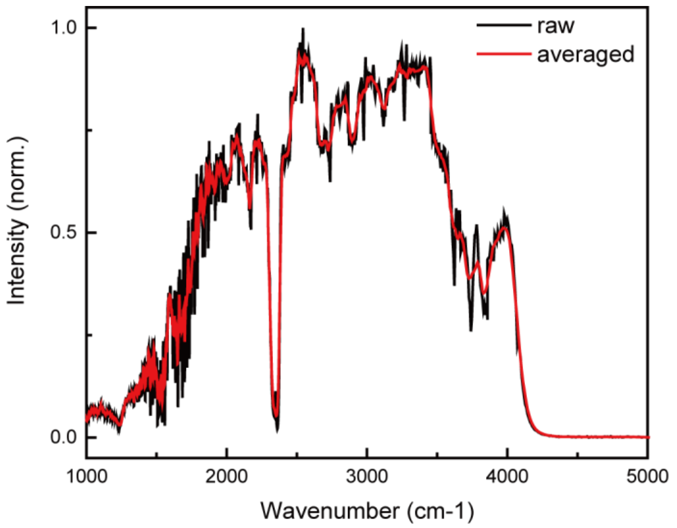

Figure S1. The raw and averaged intensities of continuum IR spectrum.

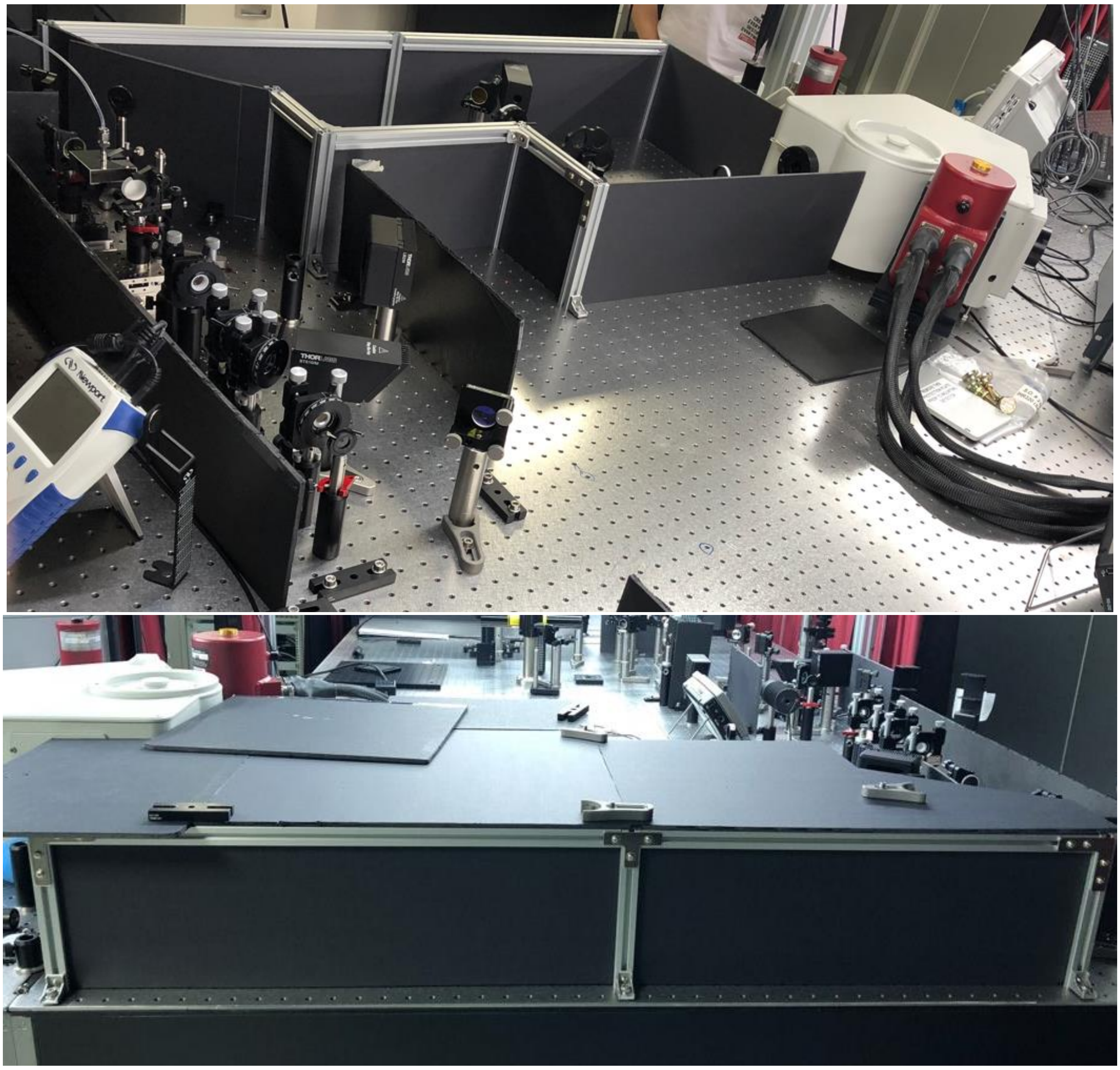

Figure S2. The captured photo of continuum IR generation setup based on plasma generation.
